# Laser- synthesized TiN nanoparticles as promising plasmonic alternative for biomedical applications

**DOI:** 10.1038/s41598-018-37519-1

**Published:** 2019-02-04

**Authors:** Anton A. Popov, Gleb Tselikov, Noé Dumas, Charlotte Berard, Khaled Metwally, Nicola Jones, Ahmed Al-Kattan, Benoit Larrat, Diane Braguer, Serge Mensah, Anabela Da Silva, Marie-Anne Estève, Andrei V. Kabashin

**Affiliations:** 1 Aix Marseille University, CNRS, LP3, Campus de Luminy, Case 917, 13288 Marseille, France; 2 Aix-Marseille Univ, CNRS, INP, Inst Neurophysiopathol, Marseille, France; 3 Assistance Publique – Hôpitaux de Marseille, Hôpital Timone, 13385 Marseille cedex 5, France; 4 Aix Marseille University, CNRS, Centrale Marseille, LMA, Marseille, France; 5 Aix Marseille Univ, CNRS, Centrale Marseille, Institut Fresnel, Marseille, France; 6 Unité d’Imagerie par Résonance Magnétique et de Spectroscopie, CEA/DRF/I2BM/NeuroSpin, F-91191 Gif-sur-Yvette, France; 7 MEPhI, Institute of Engineering Physics for Biomedicine (PhysBio), Bio- nanophotonics Laboratory, 31 Kashirskoe sh, 115409 Moscow, Russia

## Abstract

Exhibiting a red-shifted absorption/scattering feature compared to conventional plasmonic metals, titanium nitride nanoparticles (TiN NPs) look as very promising candidates for biomedical applications, but these applications are still underexplored despite the presence of extensive data for conventional plasmonic counterparts. Here, we report the fabrication of ultrapure, size-tunable TiN NPs by methods of femtosecond laser ablation in liquids and their biological testing. We show that TiN NPs demonstrate strong and broad plasmonic peak around 640–700 nm with a significant tail over 800 nm even for small NPs sizes (<7 nm). *In vitro* tests of laser-synthesized TiN NPs on cellular models evidence their low cytotoxicity and excellent cell uptake. We finally demonstrate a strong photothermal therapy effect on U87–MG cancer cell cultures using TiN NPs as sensitizers of local hyperthermia under near-infrared laser excitation. Based on absorption band in the region of relative tissue transparency and acceptable biocompatibility, laser-synthesized TiN NPs promise the advancement of biomedical modalities employing plasmonic effects, including absorption/scattering contrast imaging, photothermal therapy, photoacoustic imaging and SERS.

## Introduction

Capable of supporting collective oscillations of free electrons (surface plasmons), plasmonic nanostructures can offer a number of unique properties, including strong resonant scattering and absorption^1^, and dramatic near-field enhancement^2,3^, which makes them very promising candidates for a plethora of applications. Biomedicine looks as one of major potential beneficiaries of these plasmonic effects, provided the plasmonic materials used are compatible with biological systems^4^. Owing to its excellent chemical stability and biocompatibility, gold looks as the most suitable plasmonic material for biomedical applications, but Au nanoparticles of a reasonable small size (5–50 nm) have their plasmonic feature around 520–540 nm, which is far from optical biological transparency window located between 670 and 1000 nm. The problem of such a plasmonic mismatch can be solved by employing engineered plasmonic nanostructures such as Au-based core-shells (SiO_2_ –Au, Si-Au)^5,6^ or nanorods^7^, which shift the plasmonic feature toward the transparency window and thus enable a variety of modalities, including light induced hyperthermia-based therapy^5–7^, confocal reflectance microscopy^8^, photoacoustic tomography^9^, optical coherence tomography^10^ imaging modalities. However, the size of core-shells and nanorods typically ranges between several tens of nm and 150–200 nm, which complicates the excretion of such nanostructures from the organism^11^ and can lead to toxicity related to residual gold accumulation in some organs^12^. In addition, the nanorods are usually stabilized in colloidal solutions by non-biocompatible cetyl trimethylammonium bromide (CTAB)^13–15^, which can cause some additional toxicity problems. In general, the implementation of plasmonic feature in the biological transparency window seems hardly possible based on reasonably small NPs of classical plasmonic metals (Au, Ag, Al, etc.).

We believe that the plasmonic spectral mismatch problem of small nanoparticles can be resolved by employing alternative plasmonic nanomaterials. TiN looks to be one of most promising candidates as TiN NPs are capable of generating red-shifted plasmonic feature^16–21^ with a high photothermal conversion efficiency^21^. Having good chemical stability and biocompatibility, TiN instruments have been successfully used in biological systems as surgical and food-related tools, as well as implants^22^, while well-established surface chemistry of TiN structures renders possible its easy functionalization by standard protocols^23^. However, fabrication routes for the synthesis of TiN NPs are not always compatible with projected biomedical applications. Indeed, chemical synthesis routes^24,25^ are rather complicated in terms of a number of preparation steps and employ hazardous products, which can cause residual contamination of NPs and related toxicity issues. On the other hand, nanocrystals prepared by dry fabrication methods, including direct nitridation of TiO_2_ powders^26^, plasma assisted processing^27–29^ and laser ablation in nitrogen atmosphere^17^, normally cannot be dispersed and stabilized in aqueous solutions without applying similar wet chemistry steps.

Here, we demonstrate a simple and cost efficient method for the production of stable solutions of bare (ligand – free) TiN NPs by methods of femtosecond (fs) laser ablation and fragmentation in water and organic (acetone) solutions. By performing a series of biological tests using 2D cultures and 3D spheroids, we evidence high safety and excellent cell uptake of laser-synthesized TiN NPs, as well as show efficient therapy effect on cancer cells using TiN NPs as sensitizers of photothermal treatment at the plasmonic absorption band (650–800 nm).

## Results and Discussion

### Synthesis and characterization of TiN nanoparticles

**Figure 1:**
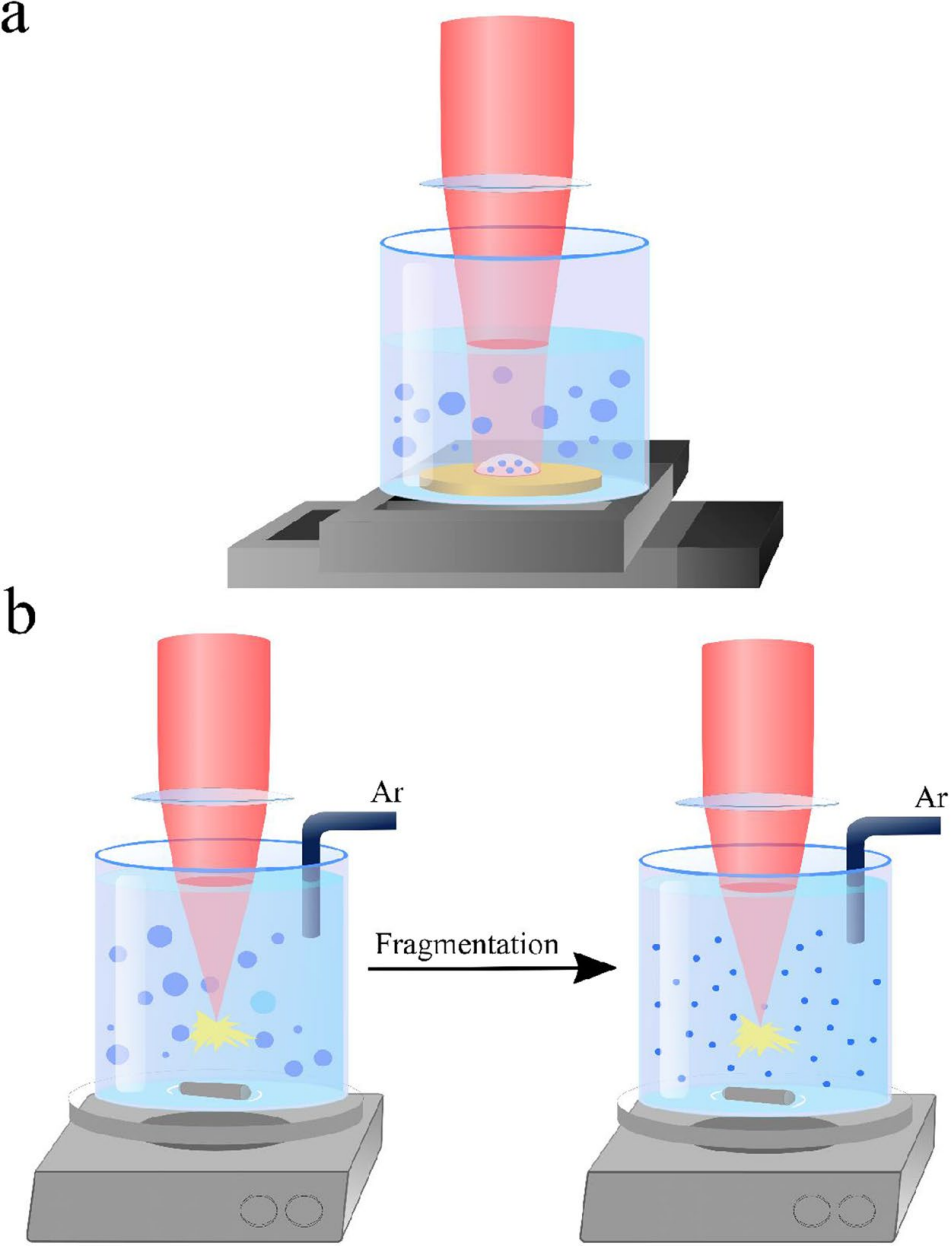
(**a**) Schematics of laser ablation setup. A laser beam is focused on the surface of the TiN target, which is placed in the vessel filled with a liquid. The vessel is mounted on a moving translation stage to avoid ablation from the same area of the target. (**b**) Schematic of laser fragmentation setup to minimize size dispersion of NPs. Ar bubbling used optionally to remove dissolved oxygen.

For the synthesis of TiN NPs we adapted methods of ultra-short laser ablation and fragmentation in liquid ambience, which were earlier used for the preparation of bare Au and Ag NPs^30–36^. In the first “ablation” approach^30–32^, radiation from a femtosecond laser (Yb:KGW, 1025 nm, 1–100 kHz) was focused onto a TiN target placed on the bottom of a glass vessel filled with deionized water or acetone in order to initiate ablation of material, as shown in Fig. 1a (see details in Methods section). The target was continuously moved at a speed of 2 mm s^−1^ during the ablation step to avoid ablation from the same point.

We observed a blue coloration of solution within a few minutes of the ablation process, indicating the formation of TiN NPs. Concentration of NPs increased linearly at the beginning of the process and then came to a saturation after some time. Saturation concentration and time until saturation depended on pulse energy. In particular, for 100 μJ pulse energy and 5 kHz repetition rate saturation time was 15 min and the saturation concentration was about 100 μg mL^−1^. TiN colloids synthesized by laser ablation both in water and acetone were very stable with almost no traces of aggregation or precipitation during their storage at ambient conditions for several months. Such a high stability of colloidal solutions was due to an electrostatic stabilization owing to strong charging of NPs surface similar to how it happens for other materials^33^. Indeed, according to our ζ–potential measurements, the surface potential of TiN NPs was −30 mV, which is higher than the stability threshold (20 mV)^37^ for colloidal solutions.

**Figure 2:**
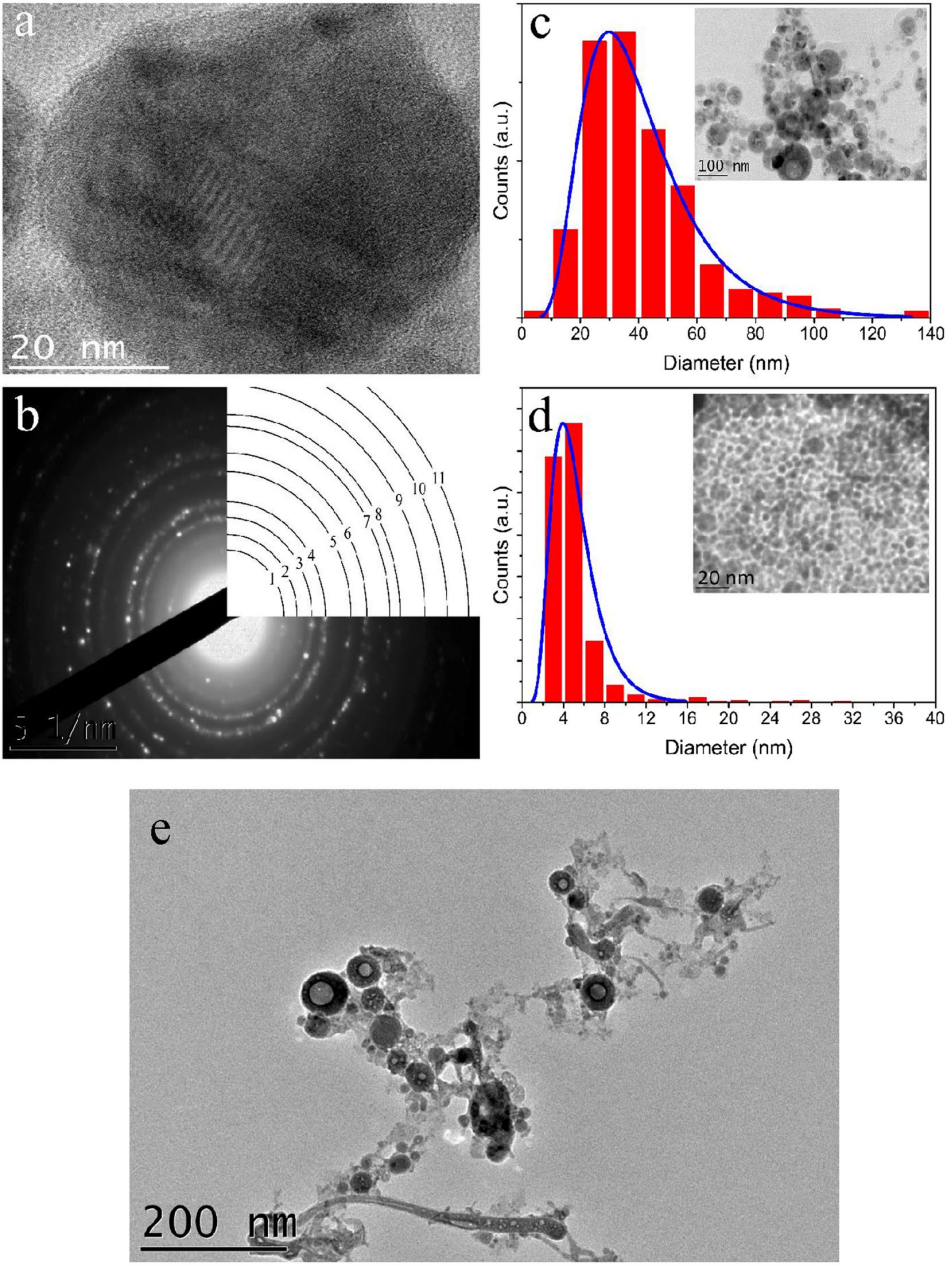
(**a**) HR-TEM image of TiN NP synthesized by pulsed laser ablation in acetone. (**b**) SAED pattern of TiN NPs with rings sequence typical for FCC lattice (see Table 1 for details). (**c**) Size distribution of TiN NPs synthesized by laser ablation in acetone at 100 μJ pulse energy and (**d**) after laser fragmentation in acetone. Insets show typical TEM images of corresponding TiN NPs; (**e**) TEM image of TiN NPs synthesized by laser ablation in water having multiple interior cavities and linked by amorphous non-spherical flakes.

A typical HR-TEM image of a TiN nanoparticle synthesized by pulsed laser ablation in water is shown in Fig. 2a. One can see that the nanoparticle had a spherical shape and exhibited a polycrystalline structure, while the size of single-crystal grains was about 1–3 nm. Interestingly, TiN NPs could have a hollow structure containing one or several cavities, as follows from the presence of light areas inside NPs on TEM images (Fig. 2e). When synthesized in acetone, only 5–10% of NPs contained such hollow cavities, while for the synthesis in water ambient the relevant parameter could reach 60%. Moreover, most of hollow NPs had only one cavity under acetone conditions, while under water conditions they could contain several cavities. As another observation, when ablated in acetone, all synthesized TiN NPs were spherical and crystalline, while the ablation in water ambient was accompanied by the production of a certain amount of nanostructured amorphous flakes. Based on our previous experience, the formation of such amorphous non-spherical flakes during laser ablation in water is typical for easily oxidizing materials (Ti, Fe, Co, etc.). Nevertheless, SAED measurements showed that spherical NPs prepared under water and acetone conditions present stoichiometric TiN. An example of SAED pattern is shown in Fig. 2b. Results of analysis of this pattern are summarized in Table 1. Rings 3, 4 and 6–11 correspond to first 8 diffraction rings of TiN FCC lattice, as follows from the comparison of their positions with data for bulk TiN, taken from JCPDS database (JCPDS 38–1420). Measured lattice constant 4.272 Å ± 0.114 Å is in accordance with tabulated lattice constant 4.24 Å of bulk TiN. Rings 1 and 5, which cannot be attributed to rock salt structure of TiN, should belong to titanium oxide fraction of synthesized NPs. In particular, position of ring 1 is close to that of (012) plane of Ti_2_O_3_ (JCPDS 43-1033), the position of ring 5 is close to (105) plane of anatase TiO_2_ and (211) plane of rutile TiO_2_ (JCPDS 21-1272 and 21-1276). Thus, SAED data clearly indicated formation of crystalline TiN NPs with lattice structure similar to that of bulk TiN with some fraction of titanium oxide. Presence of titanium oxide is in accordance with previous results on the synthesis of TiN NPs by alternative methods^20,23,25^.

**Table 1:** Values of interplanar spacings d_exp_, determined from analysis of SAED pattern of TiN NPs shown in Fig. 1b. Reference data d_hkl,ref_ of bulk TiN were taken from JCPDS database # 38–1420.

ring	d_exp_ [Å]	error [Å]	hkl	D_hkl,ref_ [Å]
1	3.708	0.100		
2	3.006	0.143		
3	2.472	0.063	111	2.449
4	2.136	0.057	200	2.121
5	1.701	0.041		
6	1.506	0.031	220	1.500
7	1.289	0.016	311	1.279
8	1.226	0.026	222	1.224
9	1.064	0.016	400	1.060
10	0.954	0.013	331	0.973
11	0.873	0.012	422	0.866

**Figure 3:**
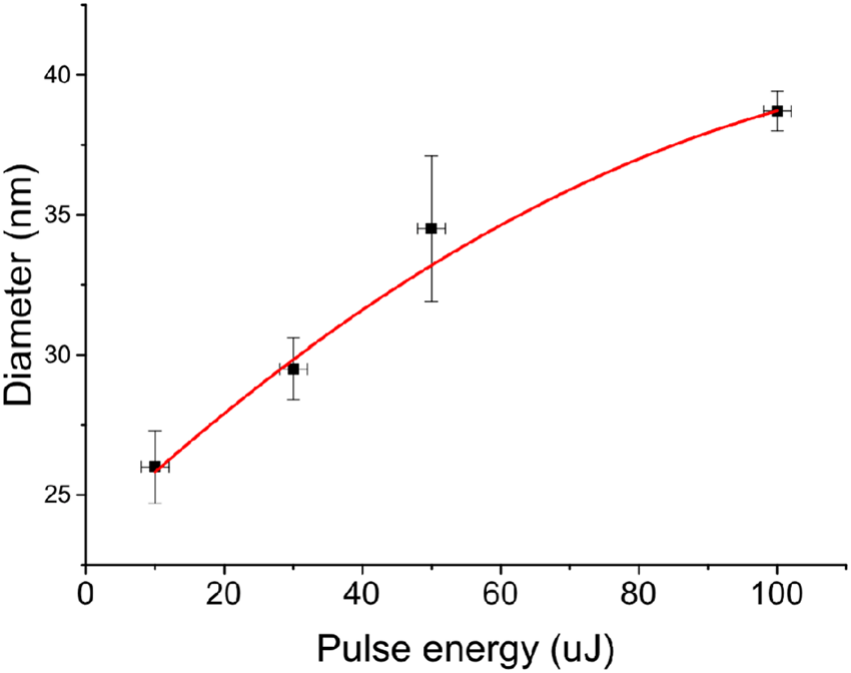
Mean diameter of TiN NPs, obtained by laser ablation in acetone at different pulse energies.

**Figure 4:**
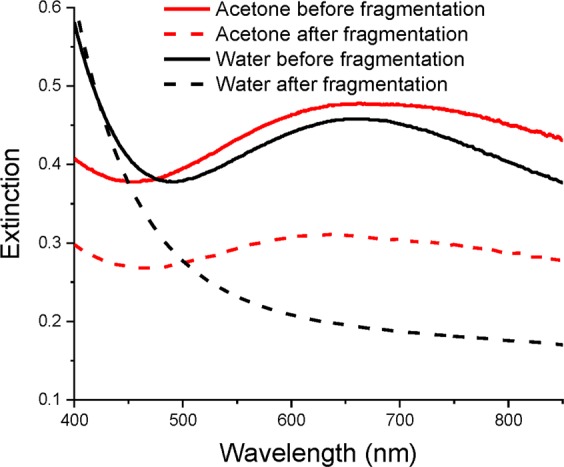
Extinction spectra TiN nanoparticles synthesized by laser ablation. Spectra showed by solid lines correspond to initial solutions of 30 nm nanoparticles prepared in water (black) and acetone (red). A corresponding size distribution of NPs prepared in acetone is shown in Fig. 2c. Spectra shown by dashed lines correspond to solutions of nanoparticles after fs laser fragmentation of initial NPs in water (red) and acetone (red). A corresponding size distribution of NPs prepared by laser fragmentation in acetone is depicted in Fig. 2d. As shown in the Figure, the mean size of TiN after fragmentation in acetone is about 5 nm.

Statistical analysis of TEM images demonstrated lognormal size distribution of synthesized TiN NPs as shown in Fig. 2c. When ablated in acetone, the mean size of NPs depended on laser pulse energy as shown in Fig. 3. In particular, the increase of pulse energy from 10 to 100 μJ led to the increase of NPs mean diameter from 25 to 40 nm under relatively broad size dispersion (25–40 nm FWHM). Such a pulse energy dependence for the mean size of TiN NPs is in agreement with our previous data on fs laser ablation of Au in deionized water^30,31^. For ablation in water, the dependence of NPs size on pulse energy was not significant and the mean size of synthesized TiN NPs was always around 30 nm under the size dispersion of 40 nm FWHM. It is important that TiN NPs prepared both in acetone and deionized water exhibited strong peaks around 700 nm in extinction spectra associated with the excitation of surface plasmons over the NPs (solid black and red lines in Fig. 4, respectively). Here, we did not observe any remarkable variations of the peak position for NPs having mean size between 25 and 40 nm. It should be noted that TiN NPs prepared in acetone could be easily transferred to aqueous solutions via a simple centrifugation step.

To further reduce the mean size and narrow down size dispersion, we employed the technique of femtosecond laser fragmentation, which was developed in our earlier works to minimize the size dispersion of bare Au NPs^34–36^. Briefly, colloidal solutions of TiN NPs, which were preliminary prepared by laser ablation step, are exposed to radiation of the same laser in the absence of the target. The laser beam was focused 1 cm below the liquid surface, while the solution was continuously homogenized by a magnetic stirrer during the fragmentation process (see details in Methods section). Here, we observed a fast loss of blue coloration of solutions and related disappearance of the plasmonic peak, while the extinction spectrum of colloids started to be similar to that of TiO_x_ (Fig. 4, black dashed line), suggesting oxidation of NPs as the main reason of the color loss. Laser fragmentation in acetone was a solution to the oxidation problem. In this case, we observed a drastic decrease in the size of final TiN NPs down to 4 nm under a very narrow size dispersion (lower than 3 nm FWHM as shown in Fig. 2d). Here, despite such a drop in NPs size, plasmon extinction peak was still well resolvable and intense, although it slightly decreased and blue shifted from 700 to 640 nm (Fig. 4, red dashed line).

The plasmonic peak around 640–700 nm for small (4–40 nm) spherical TiN NPs is much redshifted compared to Au NPs of similar and larger sizes (520–560 nm), which promises a major breakthrough in biomedical applications of nanoplasmonic structures. The extinction coefficient calculated by the Beer-Lambert law at peak position varied from 18 L g^−1^ cm^−1^ for 40 nm NPs to 6 L g^−1^ cm^−1^ for 4 nm NPs, which is comparable with other photothermal agents^38,39^. Moreover, the extinction peak was very broad and decreased by only 15% from 650 to 850 nm (Fig. 4). Such optical properties of TiN NPs look promising for their application in photothermal treatment (PTT) of tumors and photoacoustic imaging (PAI) of biological tissues.

### *In vitro* safety and cell uptake tests

**Figure 5:**
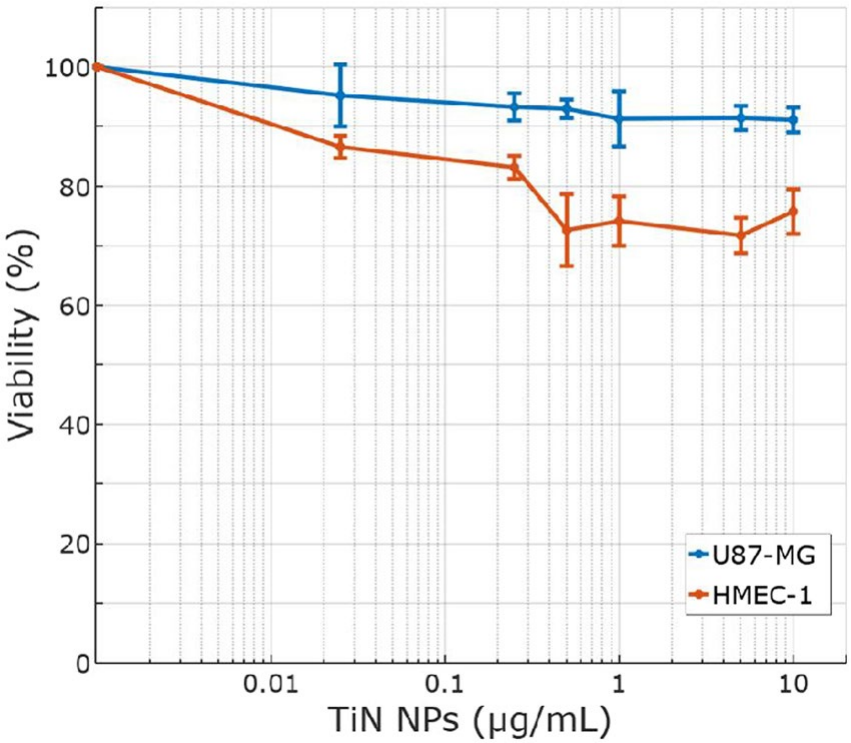
Viability of HMEC–1 (red line) and U87–MG (blue line) cells 2D cultures, assessed by resazurin reduction as a function of TiN NPs concentration.

To assess biocompatibility and cell uptake of TiN NPs synthesized by laser ablation, we performed several biological tests. Safety of new TiN NPs was tested *in vitro* on human microvascular endothelial cells (HMEC–1) and on human cancer cells (U87–MG). These cellular models in 2D and 3D geometries are highly relevant for cancer therapy applications. The viability of 2D cell cultures was evaluated on cell monolayers by the alamarBlue assay after 72 h of continuous exposure to TiN NPs with concentrations up to 10 μg mL^−1^. As shown in Fig. 5, the highest non-toxic concentration of TiN NPs for U87–MG was 10 μg mL^−1^. For all concentrations below this level the cell viability profile was higher than 90% (91 ± 3%). HMEC–1 cells survival was slightly lowered (71 ± 3%) for concentration of TiN NPs starting from 0.5 μg mL^−1^ with no further decrease at higher concentrations. The reduction of 2D cell culture viability by less than 30% and 10% for HMEC–1 and U87–MG, respectively, under relatively high concentrations (up to 10 μg mL^−1^) suggest a satisfactory low toxicity of TiN NPs. This result suggests that a special attention should be paid on vascular endothelium when toxicity of TiN is evaluated (especially in the case of an intravenous administration *in vivo*). However, in the case of intravenous injection nanoparticles are supposed to have a much reduced contact with endothelium as the NPs dose is rapidly diluted in the blood volume and distributed in different tissue compartments.

**Figure 6:**
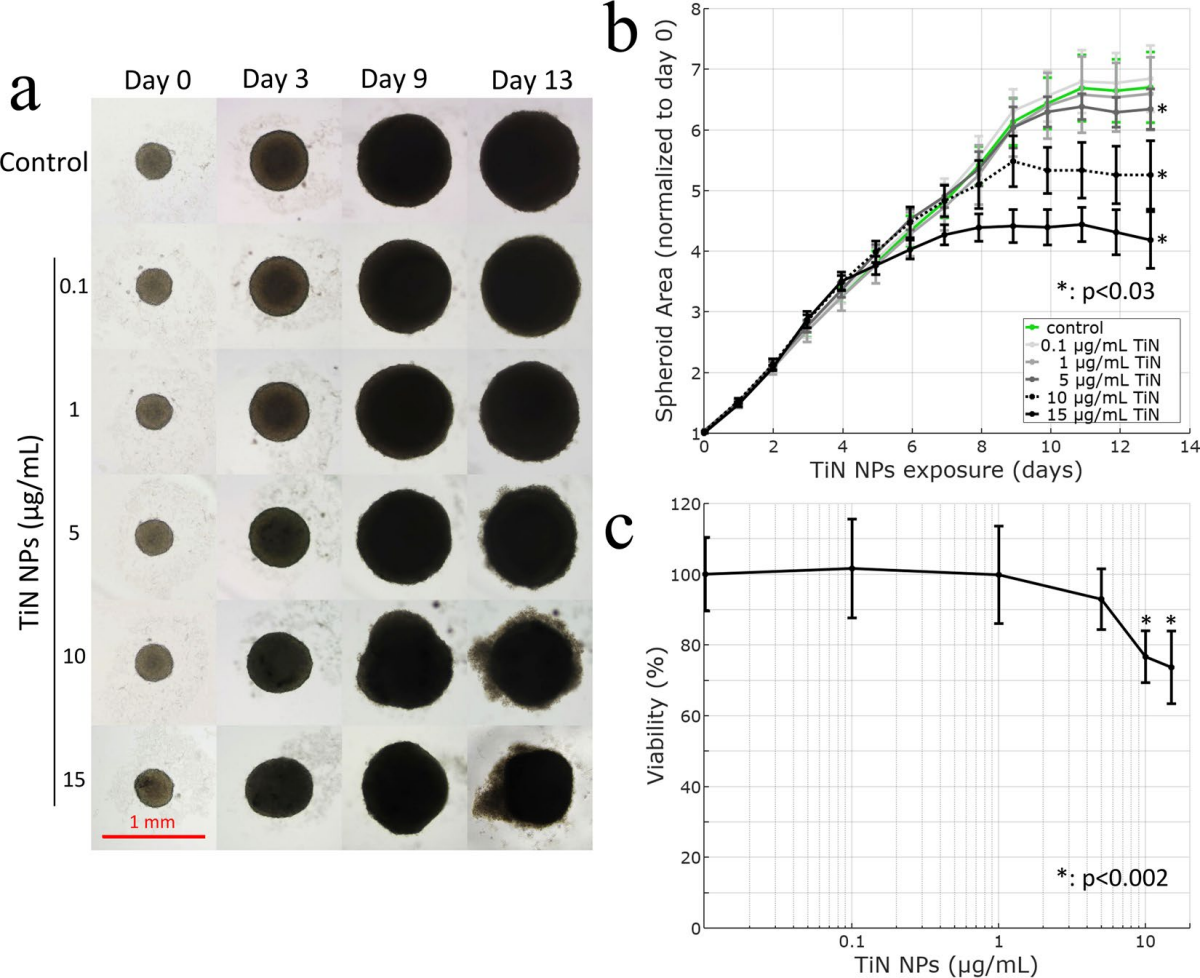
(**a**) Bright–field photomicrographs of growing spheroids of U87– MG cells exposed to different concentrations of TiN NPs, (**b**) dose- and time-dependence of spheroids area, (**c**) viability of spheroids at day 13 of their exposure to different concentrations of TiN NPs.

Similar results were obtained with 3D cell cultures in spheroids form, which are more representative of tumors-related objects as their cell morphology and phenotype are similar to those found in the original tissue architecture (tumors). Such spheroids are typically composed of clusters of cells with a dense tissue and a complex cellular organization due to absence of a culture plastic substrate. In addition, they have diffusional limits to transport of drugs, nutrients and other factors similar to *in vivo* tissues^40,41^. Safety of TiN NPs was assessed on U87–MG cells grown as spheroids and continuously exposed to different concentrations of TiN NPs for 13 days. Concentrations of 1 μg mL^−1^ and lower caused no evident morphological alterations of spheroids as compared to controls (Fig. 6a). However, after two weeks of exposure spheroids treated with doses of TiN NPs higher than 5 μg mL^−1^ exhibited irregular shapes and smaller cross-sectional areas as compared to controls. Moreover, after two weeks of continuous exposure, a loss of cell cohesion and the presence of debris were also visible for TiN NPs concentrations 5 μg mL^−1^ and above.

Quantification of spheroid cross sectional area on bright-field photomicrographs during the 13 days of exposure showed a dose- and time-dependent effect of TiN NPs on the growth of spheroids as shown in Fig. 6b. No significant reduction in cross-sectional area was observed at concentrations up to 1 μg mL^−1^, however, a gradually reduced area was observed at higher doses of TiN NPs. Indeed, at day 13 of exposure spheroids exposed to concentrations 5 μg mL^−1^ and above showed a statistically significant (t-test p values < 0.03) smaller cross-sectional area as compared to controls by 6 ± 5%, 22 ± 8% and 37 ± 8% for 5 μg mL^−1^, 10 μg mL^−1^ and 15 μg mL^−1^ respectively.

The viability assay performed on spheroids at day 13 of exposure did not show any significant toxicity of TiN NPs for concentrations up to 5 μg mL^−1^, which corresponds to the viability of cell culture 93 ± 9% as shown in Fig. 6c. Above this concentration, the decrease in cell viability was statistically significant (t-test p values < 0.002), but remained low with viability reaching 77 ± 8% and 74 ± 11% for TiN NPs concentrations 10 μg mL^−1^ and 15 μg mL^−1^ respectively. Thus, spheroids showed no morphological alterations for doses up to 1 μg mL^−1^, despite a four-fold longer exposure duration to TiN NPs as compared to 2D cultures. Moreover, viability assay performed at day 13 of TiN NPs exposure agreed with the morphological examination of spheroids as no toxic effect was observed up to a concentration of 1 μg mL^−1^. Higher concentrations of TiN NPs limited the growth of spheroids and caused slight reduction of cells viability of at most 26%. Overall, our study indicates a low toxicity profile of TiN NPs *in vitro*.

**Figure 7:**
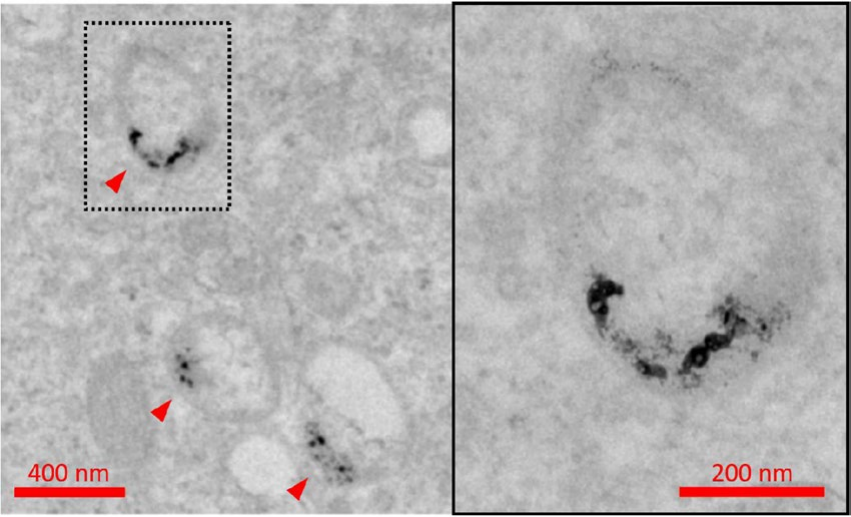
TEM images of endosomes containing TiN NPs (marked by arrows), observed after exposure of U87–MG spheroids to 1 μg mL^−1^ of TiN NPs for 10 days.

Successful plasmonic-based imaging and photothermal therapy of tumors depends on the ability of NPs to enter cancer cells. To assess cell uptake of laser-synthesized TiN NPs, we used spheroids of human cancer cells and exposed them to TiN NPs concentration of 1 μg mL^−1^ for 10 days. Then, the spheroids were examined by TEM to study the intracellular fate of NPs. Such measurements reported the presence of TiN NPs as clusters inside endosomes (Fig. 7). In contrast, our tests did not reveal any accumulation of NPs in lysosomes, nuclei, or any other intracellular structure. Altogether, these data demonstrated that TiN NPs synthesized by laser ablation can enter human cancer cells. Morphology of cell organelles including nucleus and mitohondria were normal, further confirming low toxicity effect of TiN NPs. Thus, all NPs were present as clusters inside endosomes, suggesting an active internalization via endocytosis, which is in agreement with the work of Busch *et al*.^42^, who studied chemically synthesized TiN NPs with hydrodynamic diameter 160 nm and ζ-potential −50 mV and found them in endosomes, but not in nuclei of OLN–93 cells, 3 days after the treatment of cell culture^42^. The fact that TiN NPs were observed only in endosomal vesicles and not in lysosomes could suggest that the NPs were dissolved inside lysosomes, or that they impaired the maturation process of endosomes into lysosomes.

### Photothermal therapeutic tests

**Figure 8:**
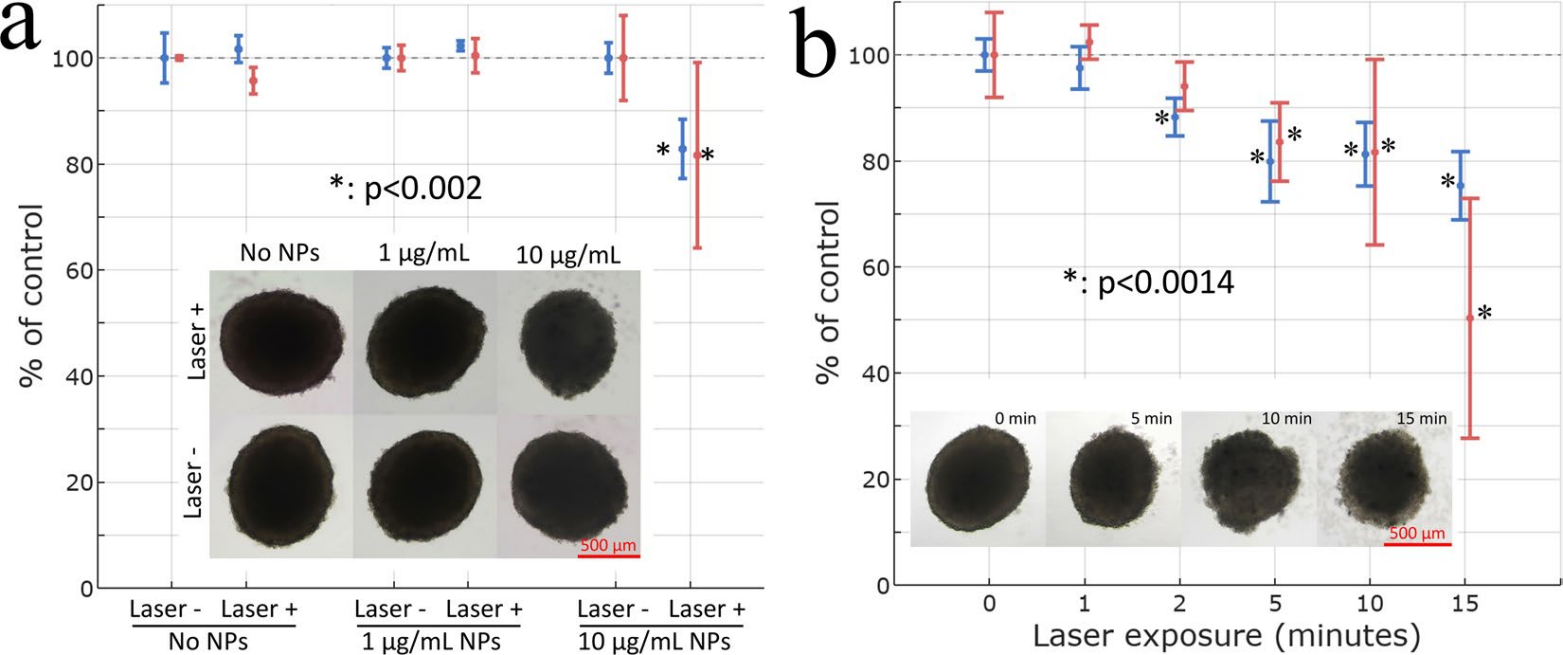
Area (blue marks) and viability (red marks) of U87–MG cells spheroids as a function of (**a**) TiN NPs concentration and (**b**) laser exposure time. Insets demonstrate corresponding bright-field photomicrographs.

A combination of good optical extinction in tissue transparency window and moderate toxicity of TiN NPs encouraged us to explore their *in vitro* photothermal activity. Spheroids of U87–MG cells were incubated with 25 nm TiN NPs and irradiated by CW laser radiation (670 nm, 4.4 W cm^−2^). Such irradiation intensity was chosen to match the relevant parameter used in earlier studies to access photothermal therapeutic activity of nanostructures^5,7,23,43^. Our tests showed that viability and growth of spheroids depended on both concentration of TiN NPs and laser irradiation time. The increase of TiN NPs concentration led to a significant reduction of both spheroids area and viability after laser irradiation for 10 min as shown in Fig. 8a. In particular, no significant effect was observed for 1 μg mL^−1^, while the increase of concentration up to 10 μg mL^−1^ led to 17.2 ± 5.6% and 18.4 ± 17.5% decrease in area and viability of spheroids correspondingly. The laser-induced effects also increased with the increase of laser exposure time, causing a noticeable reduction of spheroids area and the accumulation of cell debris after three days of incubation as shown in Fig. 8b. In particular, the area and viability of spheroids were significantly reduced as compared to controls with the highest reduction for 15 min of laser exposure (24.7 ± 6.4% decrease of area and 49.7 ± 22.6% decrease of viability).

Thus, four days of incubation with 10 μg mL^−1^ of TiN NPs and laser exposure for 10 min proved to be sufficient to induce significant toxic effect. These results outperform the photothermal effect of commercial TiN NPs obtained by He *et al*.^23^. on 4T1 cells (mouse breast tumor model). Indeed, almost no reduction of 4T1 cells viability was observed after the treatment by 12.5 μg mL^−1^ of poly(ethylene) glycol (PEG) – coated TiN NPs and 808 nm CW laser irradiation with 2 W cm^−2^ intensity for 5 min, although for HeLa cells, treated with the same parameters, 20% reduction of cells viability was recorded. Notice that TiN NPs used in their study had irregular elongated cigar-like shape with 20 nm average diameter and 100 nm length, which is much larger than renal glomerular filtration size that may lead to the accumulation of such NPs in organism.

In contrast, laser ablative synthesis results in smaller TiN NPs with sizes down to 4 nm (Fig. 2d), which may be easily cleared from the organism. Thus, laser ablative approach for synthesizing TiN NPs, described here is the versatile tool for size-controllable production of such NPs.

## Conclusions

Ultrapure TiN NPs were prepared by fs laser ablation and fragmentation in acetone and water. NPs were highly crystalline with presence of minor titanium oxide fraction. We demonstrated the possibility to tune NPs size by changing laser pulse energy, while implementation of additional fs laser fragmentation step resulted in reduction of mean NPs size down to 4 nm and significant narrowing of size distribution, which brings laser-synthesized TiN NPs to important renal glomerular filtration range (<7 nm). At the same time optical extinction spectra demonstrated a very broad plasmon – related extinction peak in near IR between 640 and 700 nm, where tissues have maximum transparency. *In vitro* tests demonstrated good internalization of TiN NPs via endocytosis and an acceptable toxicity profile of laser-synthesized TiN NPs. Finally, we demonstrated a strong photothermal effect using laser-synthesized NPs as sensitizers of IR radiation-induced hyperthermia, comparable and even outperforming commercial TiN NPs. Altogether, our results demonstrate the potential of laser-synthesized TiN NPs as a photoabsorbing agent for further photothermal studies *in vivo*.

## Methods

### Synthesis of nanoparticles

TiN NPs were synthesized by ultra-shot (fs) laser ablation of bulk TiN target in liquid medium at ambient conditions, similar to how it was done in our previous papers^30–32^. Briefly, hot-pressed TiN target (GoodFellow) was placed on the bottom of a glass vessel filled with 7 mL of ultrapure water (18.2 MΩ cm at 25 °C) or acetone (analytical reagent grade, Fisher Chemical), as shown in Fig. 1a. The liquid layer above the target was 10 mm thick. A 2.3 mm diameter beam from a Yb:KGW laser (Amplitude Systems, 1025 nm, 1–100 kHz) was focused by a 75 mm lens on the surface of the target. To account for shifts of the focal position due to non-linear effects, accompanying the interaction of intense fs pulse with liquid, we adjusted the lens – target distance to achieve maximum productivity (maximum ablated mass per a time period). This adjustment was especially important for acetone, as in the case of acetone ambience the change of pulse energy from 10 mJ to 100 mJ led to a shift of lens position by 10 mm. The target was continuously moved at a speed of 2 mm s^−1^ during the ablation step to avoid ablation from the same point.

As an additional method to control TiN NPs size we used a technique of fs laser fragmentation, which was developed in our previous works^34–36^. The experimental setup for laser fragmentation is shown in Fig. 1b. Briefly, colloidal solutions of TiN NPs, which were preliminary prepared by laser ablation step, were exposed to radiation of the same laser in the absence of the target. The laser beam was focused with the help of a 75 mm lens at 1 cm below the liquid surface. The solution was continuously homogenized by a magnetic stirrer during the fragmentation process.

To minimize oxidation effects during ablation and fragmentation processes, in some cases we pumped out oxygen dissolved in solutions by bubbling them with Ar gas before and during the experiments similarly to how it was done in our previous works^44^.

### Characterization of nanoparticles

Morphology, structure and size of synthesized NPs were characterized by the high-resolution transmission electron microscopy (HR-TEM) system (JEOL JEM 3010) operating at 300 kV and equipped with a Gatan Multiscan CCD in imaging and diffraction modes. Samples were prepared by dropping 5 μL of NPs solution onto a carbon-coated TEM copper grid and subsequent drying at ambient conditions. Analysis of selected area electron diffraction (SAED) patterns was performed using ProcessDiffraction v.8.7.1 software^45^ Uncertainty in determination of lattice constants was estimated as full width at half maximum (FWHM) of diffraction rings after lognormal correction for background signal. ζ–potential measurements were performed using a Zetasizer ZS instrument (Malvern Instruments, Orsay, France). Extinction spectra of NPs solutions were measured by a UV– VIS spectrophotometer (UV–2600, Shimadzu) using 10 mm optical path length quartz cuvettes. Concentrations of NPs solutions were determined by measuring target weight before and after the ablation step. As the TiN target was produced by hot-pressing of a TiN powder and presented a porous material, a prolonged drying step was required to determine the mass change correctly. Additional concentration measurements were done using the inductively coupled plasma – mass spectrometry (ICP–MS) method.

### Cell cultures

Safety of TiN NPs was evaluated on 2D culture of human microvascular endothelial cells (HMEC–1) and 2D and 3D cultures of human glioblastoma cancer cells (U87–MG). Cells were obtained from American Type Culture Collection (Manassas, VA, USA). HMEC–1 cells were maintained in MCDB 131 medium (Gibco, UK) containing 10% of decomplemented fetal bovine serum, 2 mM L–Glutamine (Gibco, UK), 1% (v/v) of penicillin/streptomycin solution (Gibco, UK) and 0.1% of epidermal growth factor. U87–MG cells were maintained in EMEM (Lonza, Verviers, Belgium) supplemented with 10% of decomplemented fetal bovine serum, 2mM L–Glutamine and 1% (v/v) of penicillin/streptomycin.

### Viability assay using 2D cell cultures

3000 cells for HMEC–1 and 4000 cells for U87–MG were seeded on 96 – well plates and kept at 37 °C in a humidified incubator filled with 5% CO_2_. After 24 h of growth, culture medium was replaced by 150 μL of fresh medium containing different concentrations of TiN NPs ranging from 0.025 to 10 μg mL^−1^. After a 72 h of incubation cell viability was assessed using resazurin reduction protocol: 15 μL of resazurin reagent (alamarBlue, Thermo Fisher Scientific) was added to each well, and incubated for 6 h before fluorescence was measured by a microplate reader (POLARstar Omega, BMG LABTECH). All fluorescence data were corrected by subtraction of the background signal from blank wells. Viability was calculated as a fluorescence signal from a sample, normalized to the fluorescence signal from control wells not subjected to TiN NPs. Experiments were repeated three times independently, with four wells for each experimental condition.

### Viability assay using 3D cell cultures

To obtain U87–MG cells 3D culture in a form of spheroids, cells growing in regular 2D cultures were rinsed once in phosphate buffered saline, then detached using 0.02% Trypsin/EDTA (Gibco, UK), pelleted by centrifugation at 200 g for 5 min and finally resuspended in a fresh culture medium supplemented with 20% methylcellulose and seeded on round-bottom 96 – well plates (Greiner Bio–one, Courtaboeuf, France) at 1000 cells per well in 100 μL of medium. After 72 h of growth at 37 °C under 5% CO_2_ spheroids were formed and 100 μL of fresh medium containing different amounts of TiN NPs with final concentrations from 0.1 to 15 μg mL^−1^ were added to wells. The spheroids growth was maintained for 13 days by adding 10 μL of fresh medium per well three times a week and was monitored daily by measuring spheroid area on bright–field photomicrographs (Eclipse Ts2–FL, Nikon). All images were segmented using a custom macro script, written for ImageJ software^46^. At day 13 of exposure to TiN NPs cell viability was assessed by the resazurin reduction protocol as described above for 2D cell culture, but with 20 μL of resazurin reagent per well and 18 h of incubation.

### Assay of nanoparticles cell uptake

TiN NPs uptake in spheroids of U87–MG cells was studied by transmission electron microscope (JEOL, Japan) operating at 80 kV and equipped with Megaview III camera. Six spheroids exposed to 1 μg mL^−1^ of TiN NPs for 10 days were fixed with 2.5% glutaraldehyde in 0.1 M sodium cacodylate buffer for 40 min, then washed again. Samples were progressively dehydrated with 50% to 100% ethanol bathes, before being embedded in epoxy resin (EPON 812) with a series of bathes ranging from 33% to 100% resin, and overnight polymerization at 55 °C. 60 nm sections of spheroids were obtained using Ultracut-E ultramicrotome (Reichert–Jung, USA) and contrasted by incubation in 5% uranyl acetate.

### Photothermal therapy tests on 3D cell cultures

Photothermal effect of TiN NPs was studied by exposing spheroids of U87–MG cells to different concentrations of TiN NPs for four days before laser treatment. The wells were fully filled by the culture medium and sealed with a semi-permeable membrane (Breathe–Easy, Diversified Biotech, Dedham, MA, USA) before laser exposure to avoid condensation on the microplate lids. Spheroids were irradiated by continuous-wave (CW) laser (SLD1332V, 670 nm, 500 mW, Thorlabs) with an intensity of 4.4 W cm^−2^ (310 mW power, 3 mm beam diameter) for different exposure times varying from 1 to 15 min. Spheroids area and viability were measured as described above.
